# Postmenopausal hormone and the risk of nephrolithiasis: A meta-analysis

**DOI:** 10.17179/excli2017-304

**Published:** 2017-06-30

**Authors:** Juan Yu, Binyan Yin

**Affiliations:** 1 Eastern Operation room, Yantai Yuhuangding Hospital, Medical College of Qingdao University, Yantai 264000, Shandong, P.R. China; 2 Operation room, Nanfang Hospital, Southern Medical University, Guangzhou 510515, Guangdong, P.R. China

**Keywords:** postmenopause, nephrolithiasis, hormone, women, kidney stone

## Abstract

Menopause is reported to be associated with increased urinary calcium excretion, which may enhance the risk for the development of calcium kidney stones. However, it remains controversial about whether high level of postmenopausal hormone (PMH) is a risk factor for formation of nephrolithiasis. Several observational studies have shown that PMH is protective based on 24-hour urinary parameters. Recent clinical trials provided evidence to conclude that estrogen therapy increases the risk of nephrolithiasis in healthy postmenopausal women. Our study aimed to comprehensively assess clinical evidence on the relationship between postmenopausal hormone level and risk of nephrolithiasis. To conduct systematic review, we pooled total 98 potentially related articles in Cochrane library, Medline, and Embase. Three studies with a total of 71101 study participants that included two clinical trials, 4 stratified and potentially usable results by the status of menopause and type of PMH use derived from one prospective cohort study, and one case-control studies were selected to pool relative risk using random-effect model. How the difference in menopause status, whether naturally menopausal or surgically menopausal, influenced the pooled relative risk was included in the subgroup analysis. The study population aged from 45 to 70 years old. The follow-up year and adjusted confounders differed across different studies. The pooled relative risk for the 7 stratified studies was 0.91 (95 % confidence interval (CI): [0.72, 1.14]). In the menopausal status-specific analysis, the pooled relative risk for naturally menopausal women was 0.92 (95 % CI, [0.64, 1.27]; I^2 ^= 82.74 %) whereas the pooled relative risk for surgically postmenopausal women is 0.90 (95 % CI, [0.63, 1.29]; I^2 ^= 78.47 %). The above results suggested that there was no significant association between PMH and the risk of nephrolithiasis. The difference in menopausal status did not influence the relationship between PMH and the risk of kidney stone formation.

## Introduction

Nephrolithiasis (kidney stone) is a common disease among postmenopausal women, affecting around 5 %-7 % of the population in the United States (Novak et al., 2009[16]). A sharp increase in urinary calcium stone formation after menopause implies a close correlation between hormone level and pathology of nephrolithiasis. Estrogen replacement, which is aimed at relieving symptoms of menopause, increased citrate and calcium excretion in rates in postmenopausal women with recurrent urolithiasis (Dey et al., 2002[7]). The increased stone inhibitory citrate level and increased agglomeration inhibition by estrogen replacement implied an overall beneficial and protective effect of estrogen on the risk of calcium oxalate stone formation in postmenopausal women (Iguchi et al., 1999[10]). However, recent studies found that hormone use such as estrogen replacement therapy among healthy women may be associated with a higher risk of nephrolithiasis (Maalouf et al., 2010[11]; Mattix Kramer et al., 2003[13]). The seemly-conflicted results may be due to the diverse nature of studies conducted in order to investigate how hormone is associated with the risk of kidney stone formation. Different cohort samples may also contribute to differences in the results. To the best of our knowledge, there is very few systematic review on hormone use and the risk of nephrolithiasis among healthy postmenopausal women. Here we conducted a systematic review and prospective meta-analysis on several observational studies and clinical trials to examine whether the postmenopausal hormone is a risk factor for kidney stone formation. We aimed at exploring the true effect of PMH on the risk of incident kidney stones.

## Material and Methods

### Searching strategy and selection criteria

Literature searching was performed by using Cochrane library, Medline, and Embase. The searching terms included combinations of keywords and their synonyms, such as “Nephrolithiasis”, “postmenopausal”, “Estrogen”, “kidney stone”, “renal lithiasis”, “renal stone”, “women”, “observational”, and “Clinical Trials”. And the search strategies used for the other databases were similar, with the necessary adaptions made. Additionally, we manually searched reference lists in the selected studies to identify potentially relevant studies.

### Study selection and data extraction

We set up the inclusion and exclusion criteria to include eligible studies. We focused on postmenopausal women aged around 45 to 79 years with no previous history of kidney stones and selected cohort studies that compared the risk of kidney stones among the group with PMH treatment and the group without. The estimated values of odds ratio (OR), relative risk (RR) or hazard ratio (HR) and its 95 % confidence interval (CI) needed to be specifically reported. Demographic characteristics such as age, sex, body mass index (BMI), serum calcium, and urinary calcium were described. Confounders such as age were adjusted if necessary. The exclusion criteria included: (1) only age-adjusted or other confounder-adjusted OR, RR, or HR were reported. (2) The subjects of the study had recurrent nephrolithiasis or other significant chronic kidney diseases. (3) The data were extracted from the same study population. After carefully reviewing the selected studies, the following information on the publications was collected: abstract, full text, title, author information (i.e. first name, last name, initials), and publication year. Characteristics included country of origin, sample size, age, follow-up time, type of risks and confounders-adjusted risks were extracted from the selected studies. 

### Statistical analyses

Multivariate-adjusted outcome data (ORs, RRs, HRs and 95 % CI) were extracted and converted logarithmically to log (RR) for each study. The I-squared statistic was used for heterogeneity test, and the log (RR) of the studies were pooled using fixed effect models when the heterogeneity is low (i.e., I-squared < 30%). Otherwise, a random effect model was used. Forest plots were constructed to visually demonstrate RRs and their confidence interval. Funnel plot was constructed after Trim-and-Hill correction for evaluating funnel asymmetry. Fail-safe N was used to determine the number of NULL studies that have to be added to reduce the significance of the meta-analysis to (0.05). Publication bias was analyzed by using Egger's regression test. The sensitivity analyses were conducted by omitting one study at a time to recalculate the pooled RR. All above analyses were performed by R version 3.3.2 with functional packages including ggplot2, metafor, rmeta, and meta.

## Results

### Literature search

**Figure 1:**
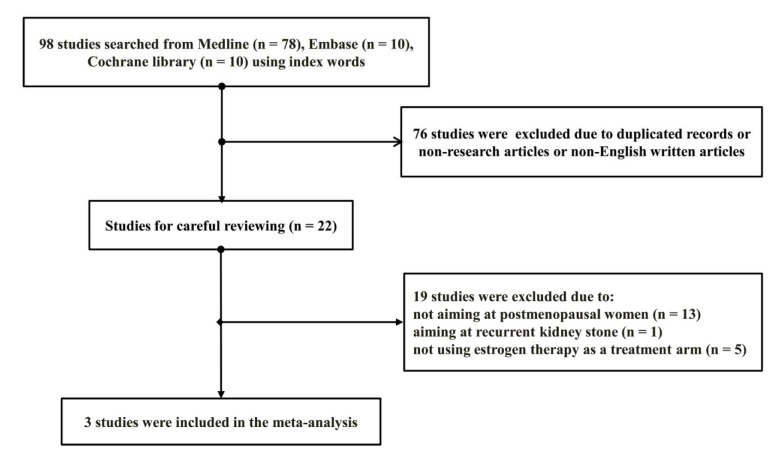
Process of study selection

A total of 98 potentially related articles were identified after employing the searching strategy in Medline (PubMed), Embase, and Cochrane library. 76 duplicated articles or non-research type's articles or non-English written articles were removed after screening their titles and abstracts. The remaining 22 articles were carefully reviewed by several independent reviewers. The selection process was demonstrated as shown in Figure 1(Fig. 1).

### Study characteristics

Three studies including one clinical study, one prospective cohort study, and case-control study were included in the meta-analysis. The characteristics of publications and their included demographics were summarized as shown in Table 1(Tab. 1) (References in Table 1: Mattix Kramer et al., 2003[13]; Maalouf et al., 2010[11]; Zhao et al., 2013[23]). Two studies were conducted in the United States and one case-control study was conducted in China. The study population was aimed at postmenopausal women with natural or surgical menopause and matched-up controls. Mattix Kramer et al. (2003[13]) studied on postmenopausal women with 26,251 undergoing natural menopause and 17,306 undergoing surgical menopause: mean age and BMI of natural postmenopausal females were 60.4 years old and 25.7 kg/m^2^, while mean age and BMI of surgical postmenopausal females were 58.3 and 25.8. The study by Maalouf et al. (2010[11]) contains two clinical trials: 10,739 postmenopausal women with hysterectomy were enrolled in the estrogen-alone trial, while 16,608 postmenopausal women without hysterectomy were enrolled in the estrogen plus progestin (E+P) trial. Women in the estrogen-alone trial were randomized to receive 0.625 mg/d of conjugated equine estrogens (CEE) or matching placebo. Women in the E+P trial were given a single tablet of CEE plus 2.5 mg/d of medroxyprogesterone acetate or matching placebo. Mean age and BMI for participants without hysterectomy in E+P trial were around 60.3 years old and 28.5 kg/m^2 ^while mean age and BMI for participants with hysterectomy in the estrogen-alone trial were around 63.6 years old and 30.1 kg/m^2^. All participants, including total 113 female patients with newly diagnosed kidney stones after menopause and 84 age frequency-matched stone-free female controls, enrolled in the case-control study conducted by Zhao et al. (2013[23]) were naturally postmenopausal: The range of age of the study subjects was 48-69 while the range of BMI of the study subjects was 15.8-32.5; Odds ratios (ORs) for associations between sex hormones indicated by the level of serum testosterone (T) and estradiol (E2) and kidney stones were estimated with logistic regression models. Potential confounding adjusted factors slightly differed across the three studies, and the primarily adjusted factors were demographic data such as age, BMI, diet, and medical history.

### Primary analysis

**Figure 2:**
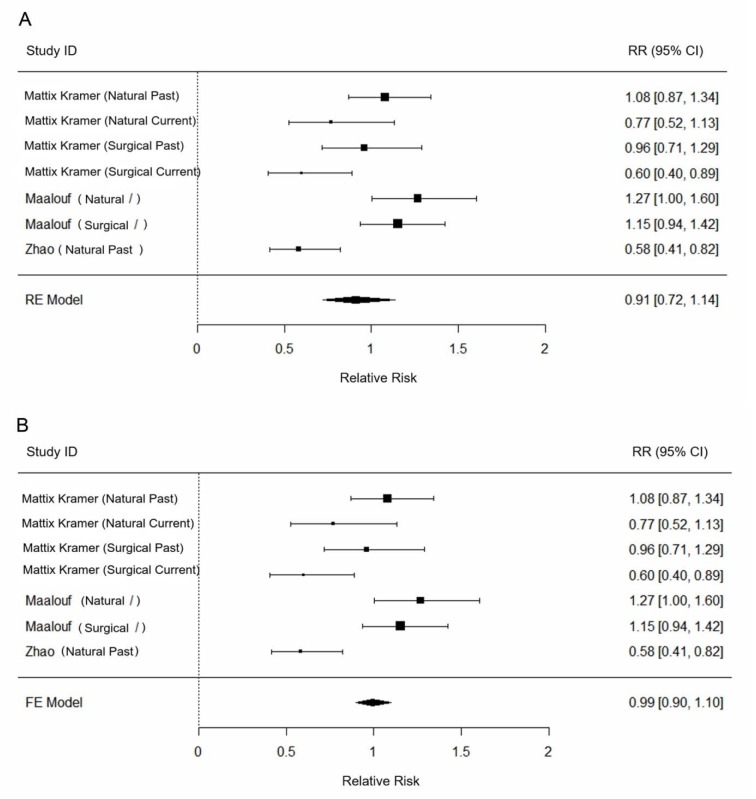
Forest plot of the association between postmenopausal hormone and incident kidney stone by random effect (RE) model (A) and fixed effect (FE) model (B).

To investigate the association between PMH and the incident of kidney stone, we first stratified the results of study population in Mattix Kramer et al. (2003[13]), into two kinds which target naturally postmenopausal and surgical postmenopausal women, in which each kind of study subjects were further divided into past users and current users with a duration ranged from 5 to 9.9 years. For the two clinical trials in Maalouf et al. (2010[11]), the results were separately analyzed. Fixed effects or random effects modeling were performed on the extracted relative risks (Figure 2A-B(Fig. 2); References in Figure 2: Mattix Kramer et al., 2003[13]; Maalouf et al., 2010[11]; Zhao et al., 2013[23]). Through heterogeneity test, we found I^2^ is 78.22 %, suggesting a high inter- and intra-study variation. Thus, a random effect modeling was used to perform the meta-analysis. According to the result analyzed by random effect modeling (**Figure 2A**(Fig. 2)), there is no significant association between PMH and the risk of kidney stone formation (the pooled relative risk: 0.91, confidence interval (CI): [0.72, 1.14], p > 0.05).

### Subgroup analysis

**Figure 3:**
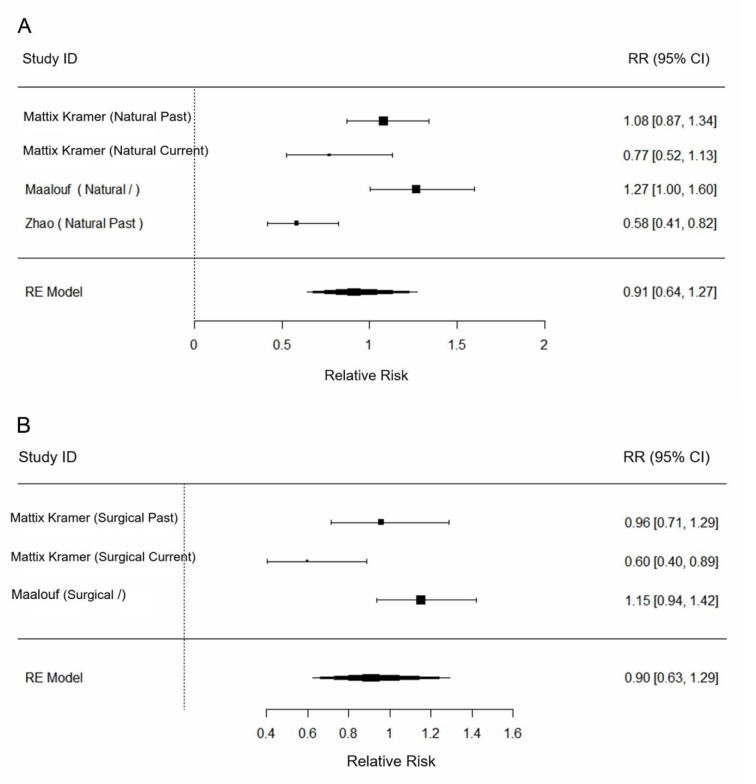
Forest plot of the association between postmenopausal hormone (PMH) and incident kidney stone by different types of menopause (A, B).

In our subgroup analysis, we aimed at investigating whether different menopause types influenced the association between PMH and the risk of nephrolithiasis. According to the results (**Figure 3A-B**(Fig. 3); References in Figure 3: Mattix Kramer et al., 2003[13]; Maalouf et al., 2010[11]; Zhao et al., 2013[23]), neither significant association between PMH and risk of kidney stone formation was found in women with natural menopause or with surgical menopause (Naturally postmenopausal women: pooled RR, 0.91; 95 % CI, [0.64, 1.27]; I^2 ^= 82.74 %; Surgically postmenopausal women: pooled RR, 0.90; 95 % CI, [0.63, 1.29]; I^2 ^= 78.47 %). 

### Sensitivity analysis

**Figure 4:**
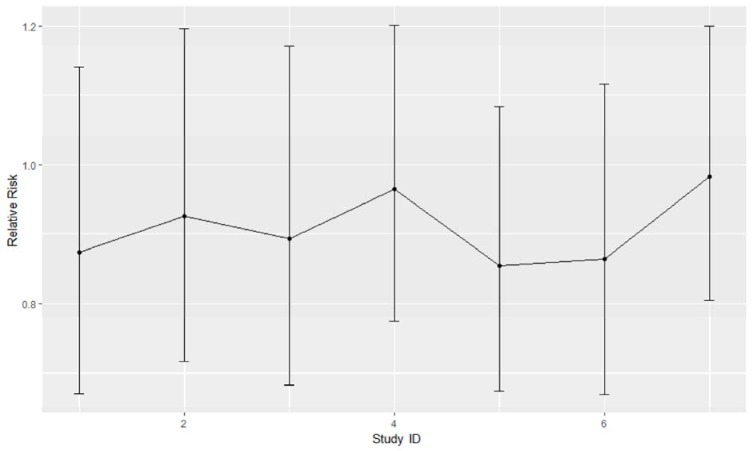
Sensitivity analysis of included publications (expressed by Relative Risk and 95 % CI)

To analyze sensitivity for each different stratified study, we conducted sensitivity analysis by omitting one stratified study and re-calculating pooled relative risk (Figure 4(Fig. 4)). Based on the result, the primary results were not influenced by omitting one study at a time.

## Discussion

In this meta-analysis including 7 stratified results and a total of 71,101 participants, we demonstrated that PMH is not associated with a statistically significant increased or decreased risk of nephrolithiasis. Different types of menopause (surgical or natural) did not contribute to identifying significantly increased or decreased risk of kidney stone.

### Publication bias

**Figure 5:**
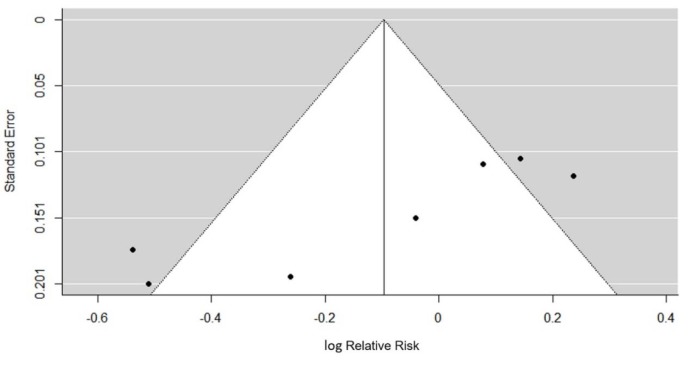
Funnel Plot of log Relative Risk against standard error with trim-and-fill correction

To investigate whether the selected studies showing a more significant intervention effect than studies with null results. We plotted Funnel plot with the Trim-and-Fill adjustment and further conducted Fail-safe N method. Null study was needed to nullify the effect. We observed in the plot that the studies with higher precision tends to report higher relative ratio. Subsequently, we performed Egger's regression test to analyze funnel plot asymmetry (Figure 5(Fig. 5)). Based on the results, we confirmed a significant publication bias (z = -4.3325, p < 0.0001), suggesting the size of the studies was associated with the reported risk ratio.

The incidence of kidney stones is increasing worldwide. Kidney stones were reported to affect approximately 1 in 11 people in the United States. The prevalence of kidney stones was 8.8 % (Scales et al., 2012[19]). Kidney stone contributes to the development of chronic kidney disease (Rule et al., 2009[18]) and confers threat of life of patients. The pathogenesis of kidney stone formation remains elusive and was influenced by the interplay of genetic and environmental factors (Coe et al., 1992[3]; Curhan et al., 1997[4]; Moe, 2006[14]). Risk factors such as dietary salt intake (Massey, 2005[12]), supplemental calcium intake (Curhan et al., 1997[5]), diabetic mellitus (Eisner et al., 2010[8]; Taylor et al., 2005[21]), beverage use (Curhan et al., 1998[6]), diet style (Taylor et al., 2009[20]), and other lifestyle factors may affect the risk of developing kidney stone. Medical management or prevention of kidney stones has gained great attention in recent decades. 

Menopause status is associated with increased urinary calcium excretion, which may increase the risk for the formation of calcium-containing kidney stones (Cappuccio et al., 2000[2]). The occurrence of kidney stone in surgically menopausal women is higher than naturally menopausal women (Mattix Kramer et al., 2003[13]). Hormone use and its effects on 24-hour urine composition have been reported in several laboratory and clinical studies. The results were inconsistent and sometimes controversial. For instance, in Sprague-Dawley rats treated with ethylene glycol, estrogen was found to appear to inhibit stone formation by increasing osteopontin expression in the kidneys and decreasing urinary oxalate excretion (Yagisawa et al., 2001[22]), suggesting a potentially protective role in the incident kidney stone. In addition, estrogen use may involve in urinary excretion of kidney stone constituents and of urinary promoters. Elevated levels of serum testosterone and serum dihydrotestosterone might be involved in increased incidences of stone formation (Gupta et al., 2016[9]). In the clinical trials conducted by Maalouf et al. (2010[11]) PMH use is associated with higher risk of kidney stone formation (Maalouf et al., 2010[11]). One possible explanation for the higher incidence of stone disease with hormone therapy (HT) could be through enhanced urinary uric acid excretion with estrogen use (Adamopoulos et al., 1977[1]; Nicholls et al., 1973[15]). Greater uric acid excretion, in turn, could lead to heterogeneous nucleation of calcium oxalate (Pak and Arnold, 1975[17]).

Our study has several limitations. Due to limited publications reporting the association between PMH and formation of kidney stone, only 3 studies were included in this meta-analysis. In the study of Mattix Kramer et al. (2003[13]) lacking information of sample size in each category by different types of PMH use and menopause results in the inability of analyzing weights for each study. Different follow-up years and specifically targeted hormones may also contribute to the difficulty in identifying the true effect of PMH on the risk of kidney stone. For instance, the estrogen-only trial used conjugated equine estrogens as treatment arm while E+P trial used estrogen plus progestin. In the study of Zhao et al. (2013[23]) they focused on measuring serum estradiol and testosterone levels in healthy postmenopausal women. Besides, we did not consider and separately analyze the incident kidney stone based on different stone components (calcium oxalate stones [COS]; non-calcium oxalate stones [NCOS]). 

## Acknowledgements

None.

## Conflict of interest

The authors report no conflicts of interest. The authors alone are responsible for the content and writing of the paper.

## Figures and Tables

**Table 1:**
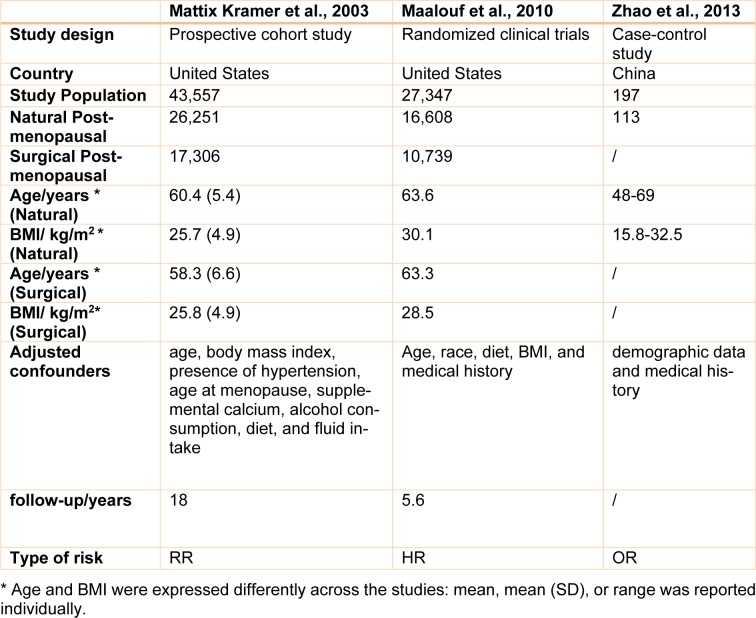
Characteristics of the selected studies
